# Establishment and validation of a predictive model of preeclampsia based on transcriptional signatures of 43 genes in decidua basalis and peripheral blood

**DOI:** 10.1186/s12859-022-05086-y

**Published:** 2022-12-07

**Authors:** Hongya Zhang, Xuexiang Li, Tianying Zhang, Qianhui Zhou, Cong Zhang

**Affiliations:** 1 Center for Reproductive Medicine, Ren Ji Hospital, School of Medicine, Shanghai Jiao Tong University, Shanghai, 200135 China; 2 Shandong Provincial Key Laboratory of Animal Resistance Biology, College of Life Sciences, Shandong Normal University, 88 East Wenhua Road, Jinan, 250014 Shandong China; 3 Shanghai Key Laboratory for Assisted Reproduction and Reproductive Genetics, Shanghai, 200135 China

**Keywords:** Decidua basalis, Decidualization, Peripheral blood, Predictive model, Preeclampsia prediction, WGCNA

## Abstract

Preeclampsia (PE) has an increasing incidence worldwide, and there is no gold standard for prediction. Recent progress has shown that abnormal decidualization and impaired vascular remodeling are essential to PE pathogenesis. Therefore, it is of great significance to analyze the decidua basalis and blood changes of PE to explore new methods. Here, we performed weighted gene co-expression network analysis based on 9553 differentially expressed genes of decidua basalis data (GSE60438 includes 25 cases of PE and 23 non-cases) from Gene Expression Omnibus to screen relevant module-eigengenes (MEs). Among them, MEblue and MEgrey are the most correlated with PE, which contains 371 core genes. Subsequently, we applied the logistic least absolute shrinkage and selection operator regression, screened 43 genes most relevant to prediction from the intersections of the 371 genes and training set (GSE48424 includes 18 cases of PE and 18 non-cases) genes, and built a predictive model. The specificity and sensitivity are illustrated by receiver operating characteristic curves, and the stability was verified by two validation sets (GSE86200 includes 12 cases of PE and 48 non-cases, and GSE85307 includes 47 cases of PE and 110 non-cases). The results demonstrated that our predictive model shows good predictions, with an area under the curve of 0.991 for the training set, 0.874 and 0.986 for the validation sets. Finally, we found the 43 key marker genes in the model are closely associated with the clinically accepted predictive molecules, including *FLT1*, *PIGF*, *ENG* and *VEGF*. Therefore, this predictive model provides a potential approach for PE diagnosis and treatment.

**Supplementary Information:**

The online version contains supplementary material available at 10.1186/s12859-022-05086-y.

## Introduction

Preeclampsia (PE) is a serious pregnancy disorder, defined as the development of hypertension and/or proteinuria after 20 weeks of gestation [1, 2]. The global incidence of PE is 5–8% of pregnancies, affecting over four million women, claiming the lives of 100,000 women and 500,000 babies each year [3]. Severe PE (sPE) can be complicated by renal, cardiac, pulmonary, hepatic, and neurological dysfunction; hematologic disturbances; fetal growth restriction; stillbirth; and maternal death [4]. To make matters worse, it increases the risk of long-term chronic diseases for both mothers and children [5]. In a Scandinavian population, women who were pregnant with sPE that necessitated preterm delivery, the risk of developing cardiovascular disease increased eight fold compared to normal puerperae [6]. However, the biological basis of this disorder is complex and multifactorial, and is not yet fully understood. Currently, the only definitive treatment for this condition is the delivery of placenta [7]. Therefore, the molecular pathology basis of PE should be explored from multiple perspectives in order to achieve early prediction and prevention.

In 2009, Redman proposed the widely accepted “two-stage model of PE” and a more refined “six-stages of PE” in 2014, in which the second stage, namely the 8th–18th weeks of gestation, is the critical period for placentation, when trophoblast cells begin to invade into the spiral arteries of the uterus [8, 9]. Once the placenta forms abnormally, PE enters the next stage [10]. This stage is most likely the key link in the development of PE, since the activation of the source signaling molecules can create a cascade amplification effect and cause sustained damage at the maternal–fetal interface [11]. Therefore, 8th–18th weeks of gestation is a prime time to search for early predictive markers of PE.

Early prevention of PE has great clinical importance for patients with PE, as it would allow clinicians to focus on high-risk groups and initiate prophylactic medical treatment. Health economists pointed out that it would be economically beneficial to screen for PE as long as effective intervention methods are available [12]. In recent years, some important progress has been made in the research of predicting PE biomarkers by analyzing changes in peripheral blood components. Low level pregnancy associated plasma protein A(PAPP-A), a glycoprotein primarily synthesized in the placenta, have been found to be associated with the development of PE in fetuses with normal chromosome number [13]. In addition, many new potential PE markers such as vascular endothelial growth factor (VEGF), placental growth factor (PIGF) [14], fms-like tyrosine kinase-1 (FLT-1) [15], endoglin (ENG) etc. are gradually being identified. These studies suggest that it is worthwhile to study the key genes of PE. In addition, other methods of predicting PE through biophysical markers are increasingly accepted. Maternal uterine artery pulsatility index (Ut A-PI), mean arterial pressure (MAP) measured at 11–13 and 19–24 weeks of gestation can predict the development and the severity of PE in some high-risk pregnancies [16, 17]. However, despite so many studies, there are still no convincing methods that can effectively predict PE. It indicates the urgency of exploring PE predicting methods.

The maternal–fetal interface is composed of decidual stromal cells, trophoblast cells and decidual immune cells, of which dNK cells account for about 70% of the decidual immune cells in the first-trimester [18]. Successful decidualization is essential for establishing and supporting a healthy pregnancy. Decidualization is a very complex gradual process, starting from the area immediately adjacent to the spiral artery of the uterus, and eventually spreading to the entire endometrium [19]. Decidualization is regulated by hormones, including progesterone and estradiol, which are ovarian steroid hormones [20–22]. After the endometrial stromal cells transform into decidual cells, they become larger and rounder in morphology, in addition, hormones, immune cells and cytokines have also undergone a series of changes. In humans, decidualization begins in the mid-secretory phase of a menstrual cycle, while in mice, this differentiation is triggered by the attachment and implantation of blastocysts [23]. More and more studies have shown that poor decidualization can lead to placental abnormalities and adverse pregnancy outcomes, which is also an important cause of PE.

One tool that can help researchers analyze the relationships between key genes and the pathogenic mechanism is weighted gene co-expression network analysis (WGCNA) [24]. Recently, the WGCNA method has been widely used due to its well-known accuracy in the biological field. Instead of linking thousands of genes to the disease, this technology focuses on the relationship between gene modules and disease traits [25, 26]. The underlying biological models of the disease can be discovered through WGCNA. In the present study, we integrated the datasets of decidualized tissues and peripheral blood, and conducted a systematic analysis. After identifying differentially expressed genes (DEGs), we performed WGCNA to find out the relationship between gene modules and disease traits in PE occurrence. We then constructed a multivariable logistic regression model based on the key signatures of this information, and constructed a predictive model for PE auxiliary diagnosis. Furthermore, we independently verified the predictive model with another two cohorts. Because the decidual defects found during delivery in women with sPE persist for at least five years after pregnancy [27, 28], indicating the essential roles of maternal factors such as decidua in PE pathology. Our results thus provide in-depth insights into the cellular and molecular mechanisms and predictive approaches that are crucial to the clinical management of PE.

## Materials and methods

### Dataset collection and identification of DEGs in PE and normal pregnancy (NP) decidua basalis

The raw datasets GSE60438 (https://www.ncbi.nlm.nih.gov/geo/query/acc.cgi?acc=GSE60438), GSE48424 (https://www.ncbi.nlm.nih.gov/geo/query/acc.cgi?acc=GSE48424), GSE86200 **(**https://www.ncbi.nlm.nih.gov/geo/query/acc.cgi?acc=GSE86200**)** and GSE85307 (https://www.ncbi.nlm.nih.gov/geo/query/acc.cgi?acc=GSE85037) were downloaded from the NCBI Gene Expression Omnibus (GEO) database. These datasets were based on the platforms of GPL6884 Affymetrix. The dataset GSE60438 contains decidua basalis samples from 25 PE patients and 23 NPs. Another dataset GSE48424 contains blood samples from 18 PE patients and 18 NPs. All the pregnant women in the cohort were between the ages of 25 and 35. The decidua basalis and blood samples were collected at delivery, with the NPs at 38–41 weeks of gestation, and the PEs at 26–38 weeks of gestation. with NPs at 38–41 weeks of gestation and PE at 26–38 weeks. The database GSE86200 contains 60 blood samples, of which 12 are from PE patients and 48 are from NPs, and GSE85037 contains 157 blood samples, of which 47 are from PE patients and 110 are from NPs. The peripheral blood samples of GSE86200 and GSE85307 were collected at 10 to 18 weeks of gestation. The details about the datasets are in the Additional files 4–7: Data 3–6. The DEGs of GSE60438 were screened by the t test method of R language, based on the *P* value < 0.05, and 9553 DEGs between the decidua of PE patients and NPs were obtained.

### Weighted gene co-expression network analysis (WGCNA)

The WGCNA R package was used to identify core DEGs associated with pathological factors in the decidua basalis between PE patients and NPs**.** The 9553 DEGs in GSE60438 with the highest expression variance and close connections were used to construct the module-eigengenes (MEs). Then, a correlation matrix was constructed using the calculated pairwise Pearson correlations between all genes. To achieve a scale-free network, β = 16 was used as the proper soft-thresholding power, the correlation between genes was exponentially calculated using the power to obtain a weighted correlation coefficient, and the pairwise correlation was then converted into an adjacency matrix of connection strength (connection strength = │correlation│^β^). To identify MEs, a dissimilarity matrix was transformed and clustered via a dynamic cut tree algorithm based on topological overlap matrix. Those preliminarily constructed modules were merged if the differences between the MEs were less than the threshold. The minimum module size was set to 300. All MEs were assigned with a unique color and also shown as a branch in the cluster tree [29]. In addition, the correlations between MEs and concerned phenotypic characteristics (in this study, disease and normal) as well as the associated significance was evaluated through module Trait Cor function.

### Annotation and enrichment analysis of gene modules

To explore the biological functions of the MEs and defined genes, gene ontology (GO) term enrichment analyses and Kyoto Encyclopedia of Genes and Genomes (KEGG) analyses were conducted. The R package “cluster Profiler” (R 4.0.3) was utilized for Gene ID conversion and GO term enrichment specification on the basis of the newest GO database version [30]. The enrichment was tested with corrections for multiple hypothesis test. The significantly enriched ontology terms were shown with bar chart according to their *P* value and classified into three categories including biological process, molecular function, and cellular component. The R package “Cluster Profiler” was also applied to assign gene sets to specific pathway maps by automatically recalling the latest KEGG online database. On this basis, the KEGG pathways were calculated and tested. The top 30 significantly enriched pathways were demonstrated by a dot plot, together with the corresponding gene numbers and enrichment scales. The input data were derived from the most MEs. The GO terms and KEGG pathways with p values less than 0.05 were considered significant.

### The logistic least absolute shrinkage and selection operator (LASSO) regression analysis

To obtain the combined results of multiple markers, we integrated and analyzed 36 blood samples from GSE48424 and the 371 core genes obtained by enrichment analysis of the MEblue and MEgrey. We used “glmnet” package (version 3.3.1) to fit the logistic LASSO regression. It is a selection method that handles the high-dimensional regression variables without a prior signature selection step, and all regression coefficients shrink toward zero, thereby forcing many regression variables to be exactly zero. We used tenfold cross-validation to select the penalty term lambda (λ) [31]. The lambda was finalized using the lambda min = 0.03087, which was the minimum mean value for cross-validated error. We via bootstrapping within the primary sampling unit and strata obtained the standard errors of the LASSO coefficients. By logistic LASSO regression analyses, we predicted and screened 43 genes most relevant to prediction from the intersections of the 371 genes and blood sample genes. We searched the STRING database (https://string-db.org/) for relationships between 43 markers. Then we obtain the hub gene networks by PPI analysis using Cytoscape_v3.8.2. For PPI, the degree is how much of protein is linked to other proteins. Taking the degree as the benchmark, the higher the degree of protein, the redder it is, and vice versa, the yellow. Moreover, a formula used to calculate the risk score (RS) of each patient, that is, the prediction model, was constructed as follows: ${\text{Risk}}\;{\text{score}} = \sum\nolimits_{i = 1}^{N} {{\text{Exp}}(i) * {\text{Coefficient}}(i)}$. In our predictive model, N (N = 43) is the number of genes, Exp is the expression value of each gene, and the coefficient is their corresponding coefficient from the LASSO regression. We would therefore be able to generate a RS for each patient, thereby classifying patients into high and low RS groups, and determining the optimal cutoff score through X-tile plots [32]. In this study, the cutoff score is 8.2973. RS > 8.2973 is the disease group, and RS < 8.2973 is the normal group.

### Receiver operating characteristic (ROC) curve analysis and the validation of the predictive model

According to the core gene coefficient of each sample, we calculated and compared the score of each sample with the actual diagnosis, and obtained the ROC diagram. The ROC curve illustrates the specificity and sensitivity of the model. Based on the expression profiles of the 43 genes, we calculated the area under the curve (AUC) for the training set (GSE48424) and different validation sets (GSE86200 and GSE85307), to assess the power of the predictive model. The closer the value of AUC is to 1, the better the model predicts. Finally, we examined the association of the 43 key marker genes in the model with well-established predictive molecules commonly used in clinical practice: fms-related tyrosine kinase 1 (FLT1), placental growth factor (PIGF), endoglin (ENG), and vascular endothelial growth factor (VEGF). Then analyzed compared their ROC curve and used a network to measure the similarity between them.

## Results

### The composition of differential genes in the decidua basalis of PE patients and NPs

**Fig. 1:**
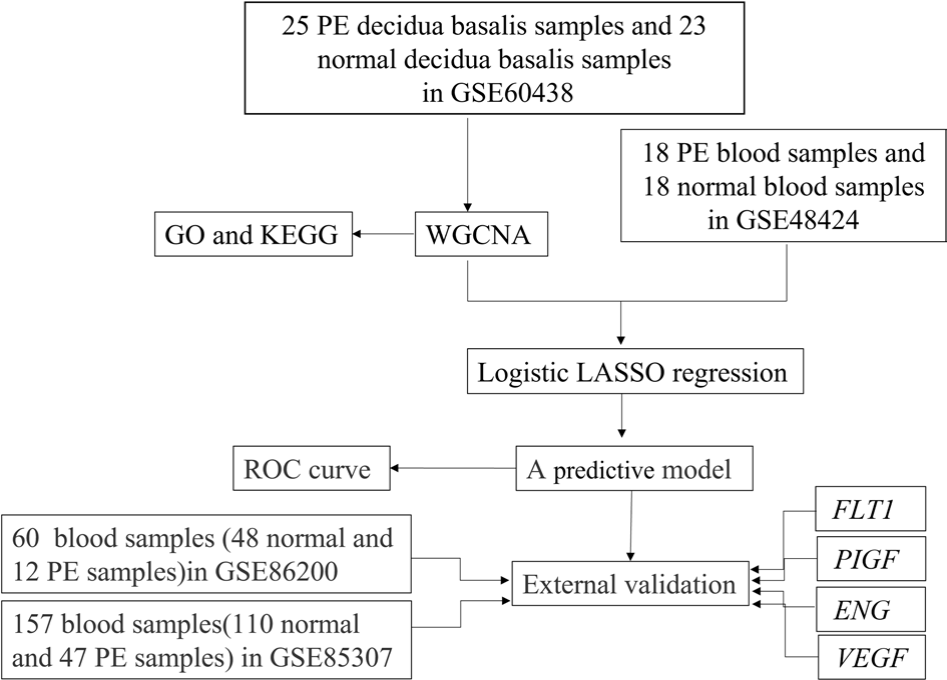
The analysis flow chart used in this study

To build the predictive model, we performed data analysis and validation, and an overview of the workflow is shown in Fig. 1. First, by analyzing the decidua basalis of 35 PE patients and 42 normal pregnancies in GSE60438, we found 9553 DEGs between the PE group and the NP group (*P* value < 0.05) for subsequent analysis. The distribution of differential genes is shown in a heatmap (Additional file 1: Fig. S1).

### WGCNA detects PE modules

**Fig. 2:**
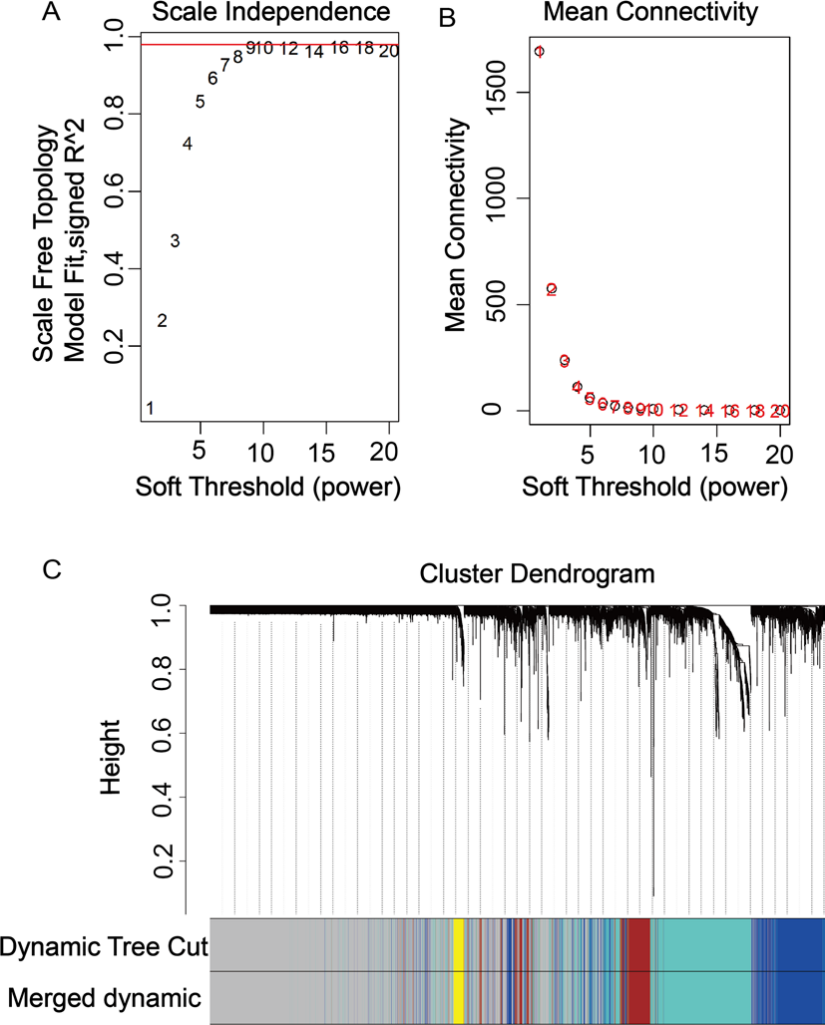
Determination of soft-thresholding power and weighted gene co-expression network analysis (WGCNA) correlation network results of preeclampsia. **A** The analysis of the scale-free fit index for various soft-thresholding powers (β). **B** The analysis of the mean connectivity for various soft-thresholding powers. **C** The cluster dendrogram, with dissimilarity determined by topological overlaps, along with assigned module colors. WGCNA can be used to group genes into five distinct module eigengenes (MEs) based on their co-expression patterns

WGCNA was performed to identify gene co-expression networks and core genes associated with the clinicopathological factors for PE. The PE dataset, namely GSE60438, was adopted from the GEO database. Notably, the soft threshold is a key parameter for WGCNA to measure genetic relationships. Here, the best soft threshold is 16 as shown in Fig. 2A, and the corresponding gene module coefficient is 16 as shown in Fig. 2B. In this regard, when the soft threshold was adjusted to a value of 16, the simulated gene network had the optimal correlation with the real biological network. After implementing a soft threshold of 16, the five most important MEs were detected. Each ME was labeled with a unique color underneath the cluster tree (Fig. 2C).

**Fig. 3:**
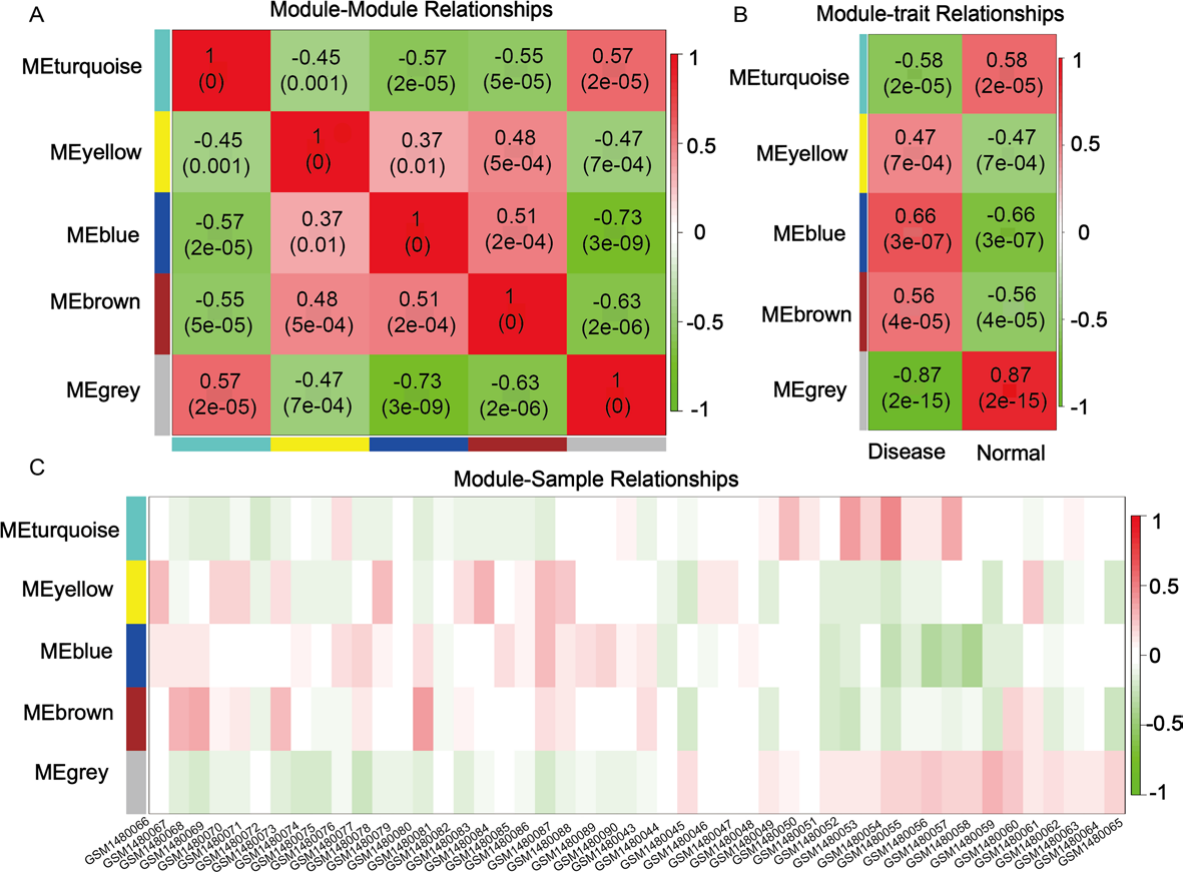
Module eigengene (ME) adjacency heatmap. MEs are defined as the first principal component of the co-expression module matrix. **A** The map shows the relatedness of the five MEs identified by WGCNA (red, positive correlation; green, negative correlation). The color scale indicates the range of the correlation coefficients. The correlation coefficient is between − 1 and + 1, where ± 1 indicates the strongest possible correlation and 0 indicates the weakest possible correlation. **B**, **C** Correlation matrix between each ME and PE severity levels. Each ME was assigned a color and was tested for correlation with the severity levels of PE (disease and normal control). The colors encode correlation coefficients (red, positive correlation; green, negative correlation). The color scale indicates the range of the correlation coefficients

For each ME, the correlation between gene expression and PE was calculated, and multiple MEs were found to be associated with PE, each was named after its representative color: turquoise, yellow, blue, brown and grey. The relationships between the MEs are shown in Fig. 3A. The results indicated strong correlations between some MEs, such as MEturquoise and MEgrey, MEyellow and MEbrown, MEblue and MEbrown, and MEblue and MEturquoise. The significance of the module-trait relations is shown in Fig. 3B. The module-sample relations are shown in Fig. 3C, from which it can be seen that MEblue and MEgrey are the most correlated, the MEblue is positively correlated with the disease group, while the MEgrey is negatively correlated with it. Moreover, the MEblue and MEgrey contain a total of 7172 genes, of which 371 core genes are most associated with PE (Additional files 2, 8: Data 1, 7).

### The enrichment analysis of GO and KEGG pathways

**Fig. 4:**
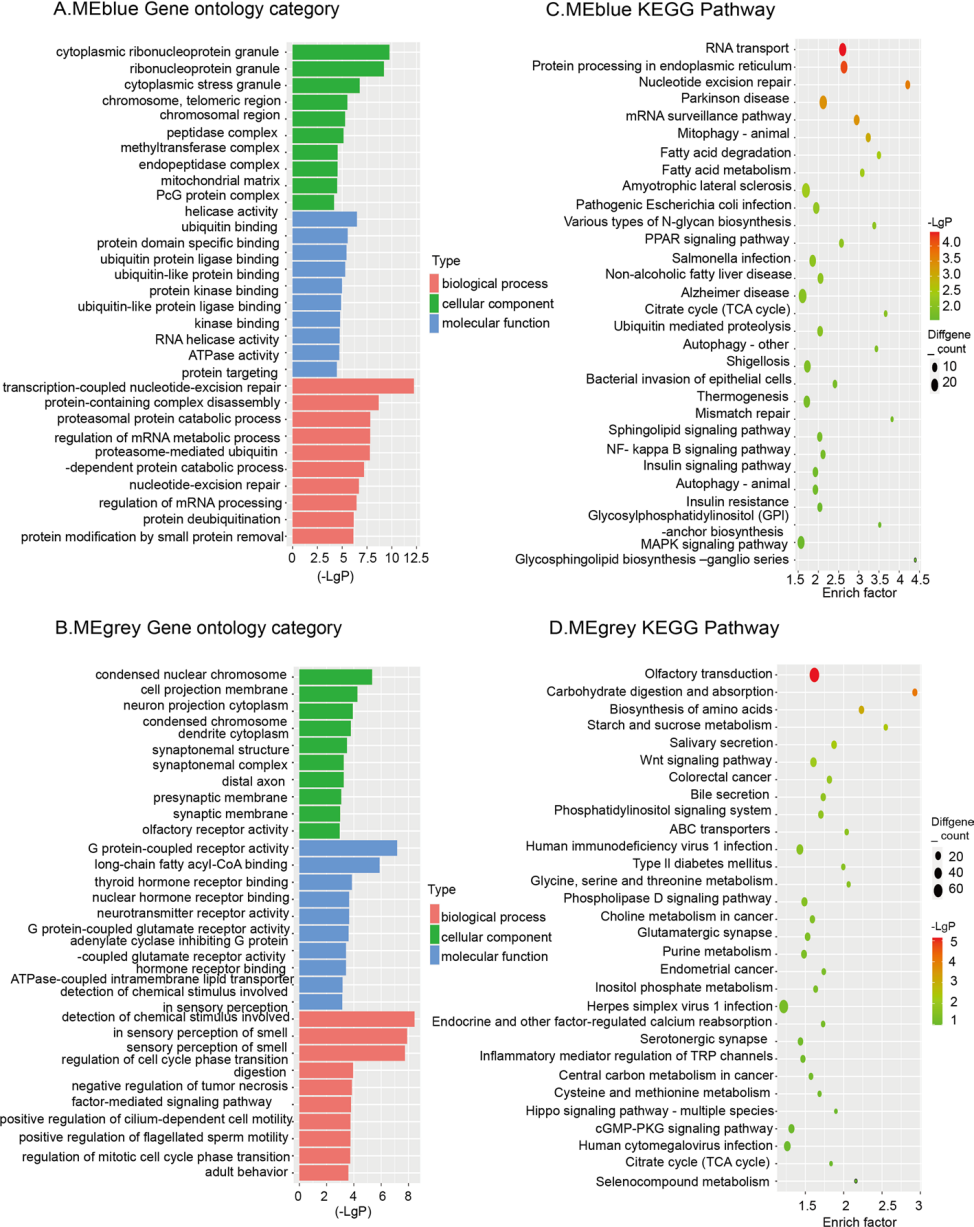
Gene ontology (GO) analysis and Kyoto Encyclopedia of Genes and Genomes (KEGG) pathway enrichment of the MEblue and MEgrey. Left panel: GO functional classification of the MEblue (**A**) and MEgrey (**B**). Green, blue, and red represent the three categories of the GO terms (cellular component, molecular function, biological process). The top 10 enriched GO terms are shown in each category. Right panel: Scatter plot for the KEGG enrichment of MEblue (**C**) and MEgrey (**D**). Rich Factor is the ratio of consensus differentially expressed genes (DEGs) annotated in a pathway to all genes in this pathway. The top 30 pathways with a *P* value < 0.05 are shown in the graph

We further analyzed the functions and the signaling pathways of the MEblue and MEgrey through GO and KEGG analysis. We performed GO analysis in terms of biological process, cellular component and molecular function to determine the gene ontology of the encoding transcripts. Subsequently, we selected the top ten GO terms and ordered them by *P* value for further analysis. In the MEblue (Fig. 4A), with regard to biological processes, the most enriched GO terms include protein targeting, transcription-coupled nucleotide-excision repair and protein-containing complex disassembly. With regard to cellular component, the most enriched GO term is cytoplasmic ribonucleoprotein granule. While the most enriched GO term for molecular function is helicase activity. Other GO terms are listed in Additional file 3: Data 2. In the MEgrey (Fig. 4B), the most enriched GO terms in the list of biological process, cellular composition and molecular function are the detection of chemical stimulus involved in sensory perception, condensed nuclear chromosome and olfactory receptor activity, respectively.

We also performed KEGG pathway analysis on the MEblue and MEgrey. In the MEblue (Fig. 4C), the five most enriched KEGG pathways associated with the PE are RNA transport, protein processing in endoplasmic reticulum, nucleotide excision repair, Parkinson disease and mRNA surveillance pathway. In addition, other pathways, such as mitophagy-animal, fatty acid degradation, fatty acid metabolism, amyotrophic lateral sclerosis, and pathogenic Escherichia coli infection, are also significantly enriched. In the MEgrey (Fig. 4D), olfactory transduction, carbohydrate digestion and absorption, biosynthesis of amino acids, starch and sucrose metabolism, and salivary secretion are the top five pathways, followed by Wnt signaling pathway, colorectal cancer, and bile secretion [33–35]. Furthermore, the phosphatidylinositol signaling system, ABC transporters, and human immunodeficiency virus 1 infection were also significantly enriched. Overall, these results indicate that the MEblue and MEgrey might play a vital role in the decidua.

### Construction of predictive model and establishment of 43 gene expression signatures

**Fig. 5:**
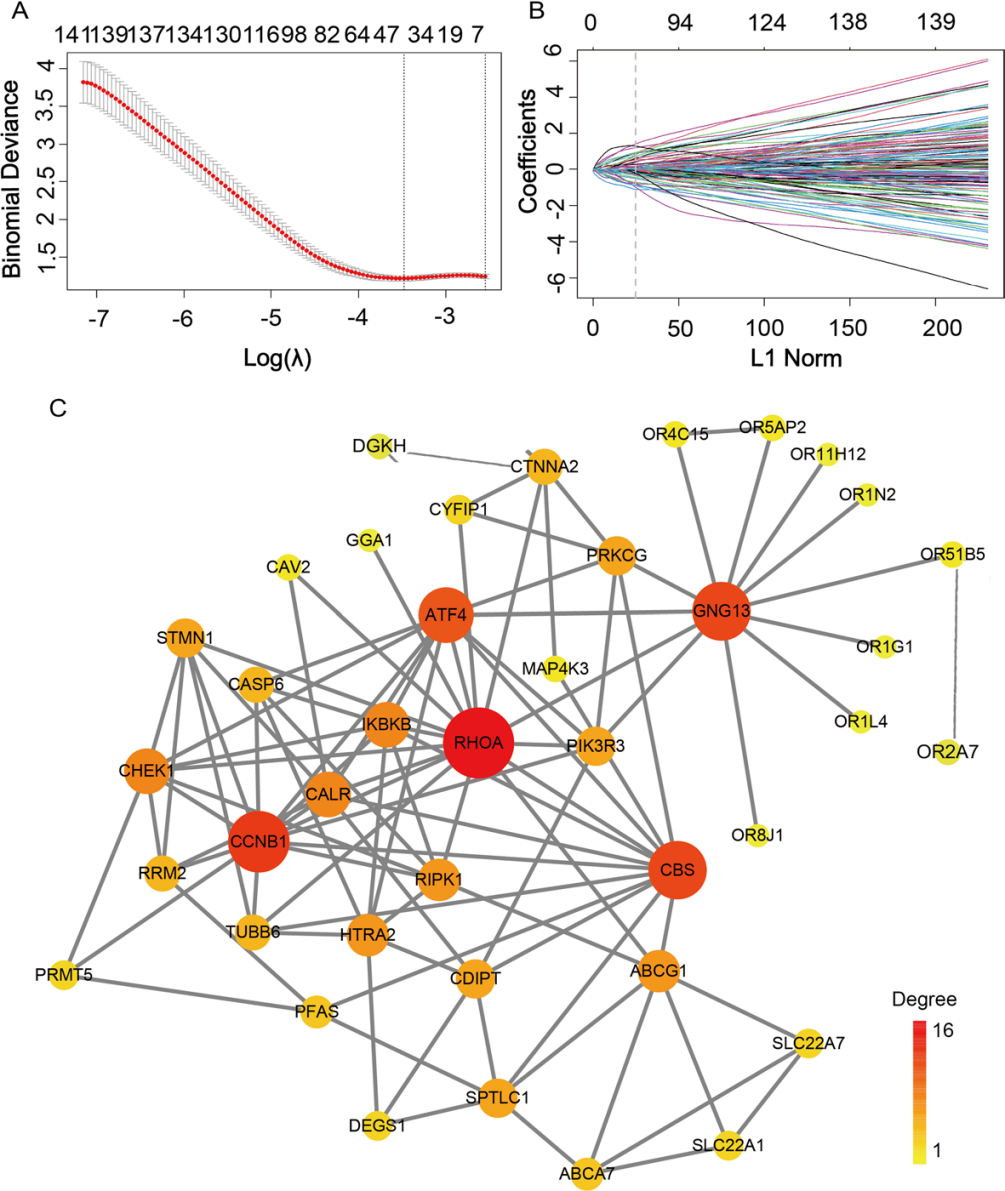
Selection of the genes by the logistic least absolute shrinkage and selection operator (LASSO) regression. **A**, **B**. LASSO coefficient profiles of the 371 differentially expressed associated genes. Each curve corresponds to one gene; the vertical line is drawn at the value lambda = 0.03087 chosen by tenfold cross-validation. **C** The network interaction of the 43 gene set inferred using the IMEx Interactome Database. Red nodes indicate the proteins present in the 43 gene set. The size of the node is proportional to the number of connections. The large nodes represent a few highly concentrated hub nodes, while most small nodes have only a few connections

We then used logistic LASSO regression analysis to intersect the 371 core genes from the MEblue and MEgrey with blood sample genes to screen for co-expressed genes and constructed predictive model. The trend of the LASSO coefficients is shown in Fig. 5A. We selected the best 43 markers with the lowest error rate, as shown in Fig. 5B. The coefficients for these 43 markers are shown in Table 1. We constructed the evaluation formula, which is the predictive model: ${\text{Risk}}\;{\text{score}} = \sum\nolimits_{i = 1}^{N} {{\text{Exp}}(i)*{\text{Coefficient}}(i)}$. Subsequently, we explored the underlying biological network of these 43 candidate genes, we used the 43 genes as seeds to generate a minimal interaction network, as shown in Fig. 5C. The network includes 40 of the 43 genes and is centered on basic nodes such as *GNG13*, *RHOA*, *CBS*, *ATF4*, *CCNB1*, *CALR*, *IKBKB* and *CHEK1*.

**Table 1:** The 43 genes used to determine the predictive model

Gene	Coefficient
ABCA7	0.541868753
ABCG1	− 0.06517424
ATF4	0.838095044
CALR	0.031047572
CASP6	0.146229425
CAV2	− 0.180641438
CBS	− 0.059166669
CCNB1	− 0.042959353
CDIPT	− 0.808012575
CHEK1	− 0.002422351
CTNNA2	0.055547405
CYFIP1	− 0.107809352
DEGS1	− 0.111781866
DGKH	0.136330674
GGA1	0.024137534
GNG13	0.15972311
HTRA2	− 0.174892988
IKBKB	0.158093266
MAP4K3	− 0.425954731
OR11H12	0.461050481
OR1G1	− 0.134170642
OR1L4	0.001125134
OR1N2	0.786152706
OR2A7	0.20659641
OR4C15	0.446657402
OR51B5	0.282437104
OR5AP2	− 0.164222624
OR8J1	− 0.548971209
PDE11A	− 0.300761564
PFAS	− 0.271570254
PIK3R3	− 0.014831635
PPT2	0.024398568
PRKCG	0.190530227
PRMT5	− 0.070152948
RHOA	− 0.492044815
RIPK1	1.186303398
RRM2	− 0.000843929
SLC22A1	− 0.202447713
SLC22A7	− 0.554879551
SLC4A5	0.082317787
SPTLC1	− 0.454811615
STMN1	− 0.032054443
TUBB6	0.600532672

### Validation of the predictive model

**Fig. 6:**
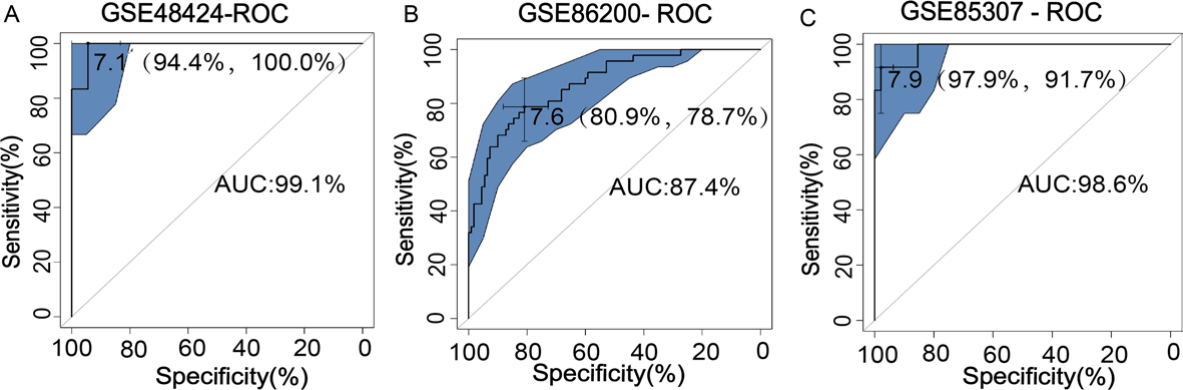
Receiver operating characteristic (ROC) curve analysis of both the training set and validation sets. ROC curve of the model from training set of GSE48424 (**A**). ROC curves of the model from validation sets of GSE85307 (**B**) and GSE86200 (**C**)

We verified the specificity and sensitivity of the predictive model by ROC curve analysis. We obtained an AUC of 0.991 and a C-index of 0.991 [94.4%, 100%] for the training set by ROC analysis (Fig. 6A). The AUC of the subjects is close to 1, indicating that the prediction results are reliable, and the predictive model has a good predictive effect. The validation set contained a total of 60 blood samples in the GSE86200. The AUC of GSE86200 is 0.874 [80.9%, 78.7%] by ROC analysis (Fig. 6B). Another validation set includes a total of 157 blood samples in GSE85307. The AUC of GSE85307 is 0.986 [97.9%, 91.7%] by ROC analysis (Fig. 6C). Therefore, our predictive model based on the analysis of decidual tissue and peripheral blood has good stability, indicating that the predictive model is reliable and credible.

**Fig. 7:**
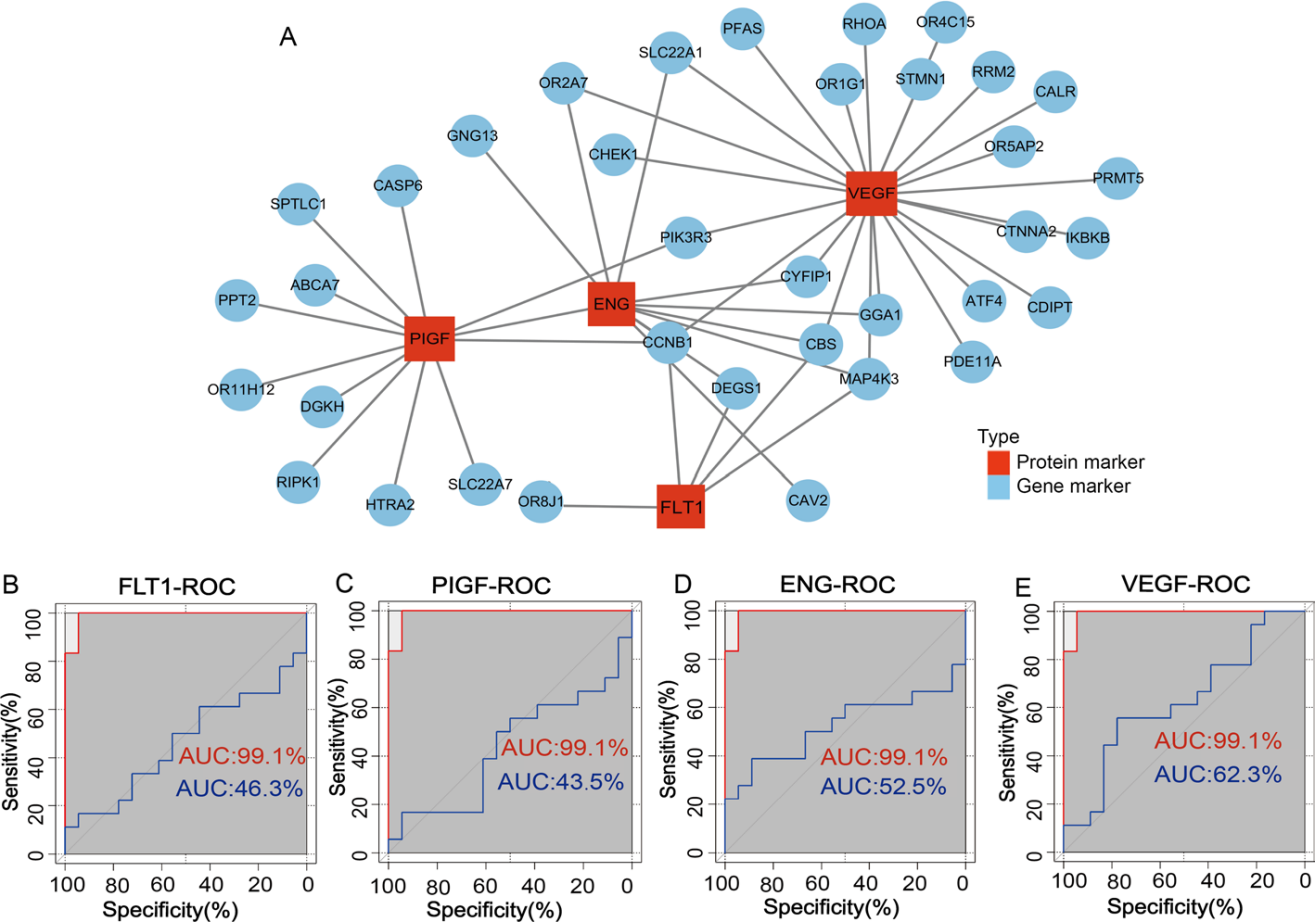
The validation of the predictive model. **A** Modeling the correlations between the screened makers and clinically commonly used markers fms-related tyrosine kinase 1 (*FLT1*), placental growth factor (*PIGF*), endoglin (*ENG*) and vascular endothelial growth factor (*VEGF*), *P* value < 0.05. The ROC curve analysis showed an AUC of 46.3% for *FLT1* (**B**), 43.5% for *PIGF* (**C**), 52.5% for *ENG* (**D**), and 62.3% for *VEGF* (**E**)

In order to understand the correlation between the screened core genes and some commonly used clinically predictive molecules including *VEGF*, *PIGF*, *FLT1* and *ENG*, we made their correlation network diagrams after analysis (Fig. 7A). We found that 36 core genes were associated with these canonical molecules. In addition, we tested these molecules with ROC analysis and the results showed that the AUC of *FLT1* is 46.3% (Fig. 7B), *PIGF* is 43.5% (Fig. 7C), *ENG* is 52.5% (Fig. 7D), and *VEGF* is 62.3% (Fig. 7E), respectively.

## Discussion

In this study, we used conventional expression profiles to compare the DEGs expressed in the decidua basalis of the PE group and NPs. We searched for co-expressed MEs and core genes through WGCNA, and explored the association of MEs with PE. We found that among the five most significant MEs obtained, the correlation between MEblue and MEgrey had significant differences in the correlation between the disease group and the normal group. MEblue was positively correlated with the disease group, while MEgrey was negatively correlated with the disease group. We further selected the 43 most relevant markers and built a predictive models using logistic LASSO regression analysis combining the core genes from MEblue, MEgrey and blood samples. The validation sets revealed good performance of the predictive model. Finally, we found the 43 key marker genes in the model were shown to be closely associated with the generally accepted predictive molecules, including *FLT1*, *PIGF*, *ENG* and *VEGF*. Therefore, bioinformatics analysis is a feasible strategy to investigate PE predictive models for PE prevention.

Most previous studies on the pathogenesis of PE have focused on placenta, including placental ischemia and placental dysfunction, while ignored the effects of decidua. Therefore, most of the marker molecules predicting PE are from the placenta. However, endometrium is the basis of placentogenesis and growth. More and more studies have shown that abnormal decidualization is an important factor leading to the occurrence of PE [27, 36, 37]. A large number of decidualization-related genes are abnormally expressed in the decidua of PE patients [38–43]. The transcriptional signatures that promote endometrial decidualization deficiency can be detected before or after pregnancy [28, 44, 45]. In this study, we analyzed the expression profiles of decidual tissues, identified a number of genes associated with PE. Further analyzing the DEGs with GO and KEGG, we found that biological processes are significantly enriched in multiple biological processes such as proteasomal protein catabolic process, RNA splicing, protein deubiquitination, histone modification. The significantly enriched pathways are PPAR signaling pathway, RNA transport, carbohydrate digestion and absorption, biosynthesis of amino acids, and starch and sucrose metabolism. These results suggest that a variety of biological processes and signaling pathway play import ant roles in decidualization.

We identified 43 highly expressed markers by analyzing the mRNA levels of the blood samples. We found that *CCNB1*, *RHOA*, *ATF4*, and *CBS* represent the most highly interacted hub genes, suggesting their vital roles in PE pathogenesis. Previous studies have demonstrated that CCNB1, a member of the cyclin family, is particularly critical for the maintenance of the mitotic state [46]. The overexpression of CCNB1 leads to unscheduled cell cycle entry, uncontrolled cell proliferation and tumorigenesis [47]. RHOA has been investigated as an essential molecule involved in signal transduction and the regulation of gene transcription, thus affecting physiological functions such as cell division, survival, proliferation and migration [48]. ATF4 is a stress-induced transcription factor that is frequently upregulated in cancer cells. ATF4 controls the expression of a wide range of adaptive genes, enabling cells to withstand stress, such as hypoxia or amino acid limitation [49]. CBS-deficient patients are prone to vascular thrombosis. Studies have shown that lack of CBS results in blood coagulation defects, underlying its high susceptibility to vascular thromboembolism (50% chance at the age of 30), which is the major cause of morbidity and mortality [50]. However, the effects of these genes on PE remain unknown. Here, our study found the expression of CCNB1, RHOA, ATF4 and CBS were significantly changed in PE compared with normal tissues, indicating that their abnormal expression may contribute to disorders in cell cycle, cell migration and coagulation function in PE patients, suggesting they are essential for PE progression and are likely to be novel PE markers.

In this study, we assembled a large number of GEO database, pioneered the combined analysis decidual tissue and peripheral blood, and explored a model for predicting PE using cutting-edge bioinformatics techniques. In order to verify the stability of the model, another two groups of samples were randomly selected for verification. Encouragingly, the results matched expectations, with the AUCs exceeding 80% for both validation sets. Previous studies have shown that some candidate molecules, including FLT-1, PIGF, ENG and VEGF, can be used to predict PE by analyzing the changes in maternal peripheral blood composition [14, 51]. It has been shown that the circulating levels of ENG increase the predictive value of the ratio of sFlt-1 to PlGF in maternal serum in the diagnosis of both term and preterm PE [52]. Studies have also shown that PLGF and VEGF play critical roles in fetal angiogenesis during pregnancy. Placental hypoxia stimulates the production of these angiogenic factors and their endogenous inhibitors, and the imbalance of the production of these factors may lead to PE [53–55]. However, when we correlated the above four molecules (FLT-1, PLGF, ENG and VEGF) with these 43 markers and analyzed their ROC curves, we found that although there were strong correlations between them, the AUCs are less than 80%.

In order to answer this question, after analysis, we found that although the expression levels of these molecules commonly used to predict PE clinically are significantly different between PE group and the control group, they have some limitations. A large number of studies have shown that single molecules cannot accurately predict PE. As a single biomarker of PE, PPAP-A only predicts 22% of the PE cases in the first trimester of pregnancy with a false positive rate (FPR) of 5%. When combined with Doppler ultrasound uterine artery measurements, the prediction rates (PR) increases to 32% at 5% FPR [56]. PIGF has been shown to be less expressed in early PE pregnancies, as a single biomarker, PIGF has a PR of 47% at 5% FPR [57]. Therefore, there has been growing interest in studying combinatorial approaches for the prediction and prevention of abnormal outcomes based upon multivariable models to improve the sensitivity and specificity [58–60]. Researchers have examined a significantly elevated SFLT-1/PlGF ratio in patients with PE caused by placental insufficiency [61, 62]. Zhang et al. combined circRNA with ENG to enhance the predictive power for early PE [63]. Another reason is that the above-mentioned molecules commonly used in clinical prediction are proteins, while our model is constructed based on the RNA level of the screened markers. In addition, the monitoring of protein level and vital signs has a certain lag, while the detection of RNA molecules is a more rapid, convenient and accurate way [64], which can greatly improve the time point of PE diagnosis and effectively extend the window period for PE prediction and treatment. Therefore, the low values of individual protein candidates in the predictive model reflect the limitation of a single protein molecule in predicting PE.

Here, we analyzed the decidual tissue of postpartum patients in conjunction with peripheral blood to find more direct and effective predictive molecules. We identified 43 hub genes as candidate biomarkers for PE and built a predictive model. These hub genes may provide a theoretical basis for targeted therapy against PE. Predicting from mRNA levels is expected to be a more effective prediction method for PE prediction as well as diagnosis and treatment. Recently, Rasmussen and colleagues also found that maternal plasma cell-free RNA (cfRNA) can predict PE and preterm birth during the asymptomatic stage. Using a large number of blood samples, they found that the cfRNA pattern-based analysis achieved a positive predictive value of 32% with a sensitivity of 75% regardless of clinical factors such as maternal age, body mass index, and ethnicity [65], which is quite convenient and easy to carry out. In our study, we used clinical data from decidua tissue and blood samples combined with machine learning to provide more clues for discovering hub genes and developing predictive models. The results on the two validation sets found that the AUCs of our predictive model are 0.874 and 0.986, and the sensitivities are 78.7% and 91.7%, respectively.

Our study has some limitations. First, the pathogenesis of PE is complex with many influencing factors. Here, we analyzed the changes in decidua and peripheral blood, which may not reflect all the influencing factors. Second, the onset period of PE is not easy to determine, since maternal decidual tissue can only be collected at the time of delivery, which cannot represent the entire course of PE, it is also an insurmountable problem encountered in the entire PE research field. Third, the limited patient groups. As these data come from different research institutes with different research purposes and contents, it is difficult to unify them. Despite the above limitations, to our knowledge, this is the first study using WGCNA to identify the key modules in PE and provide novel biomarkers for prediction. In future work, molecular biological experiments and/or cytometry analyses are required to verify these findings, and another external verification based on larger samples should be conducted.

## Supplementary Information

**Additional file 1: Fig. S1.** The heatmap shows the distribution of differentially expressed genes.

**Additional file 2: Data 1.** The MEblue and MEgrey contain genes

**Additional file 3: Data 2.** MEblue Go terms

**Additional file 4: Data 3.** The raw clinical data of GSE60438

**Additional file 5: Data 4.** The raw clinical data of GSE48424

**Additional file 6: Data 5.** The raw clinical data of GSE86200

**Additional file 7: Data 6.** The raw clinical data of GSE85307

**Additional file 8: Data 7.** The 371 key genes

## Data Availability

Publicly available datasets were analyzed in this study. The datasets (GSE60438, GSE48424, GSE86200 and GSE85307) supporting this study can be found in the GEO (https://www.ncbi.nlm.nih.gov/geo/). Raw data is available in the Supplementary Files.
