# Supplementary figures and images for: Establishment and validation of a predictive model of preeclampsia based on transcriptional signatures of 43 genes in decidua basalis and peripheral blood
Source: BMC Bioinformatics. 2022 Dec 7;23:527. doi: 10.1186/s12859-022-05086-y (PMC9730617; doi:10.1186/s12859-022-05086-y)

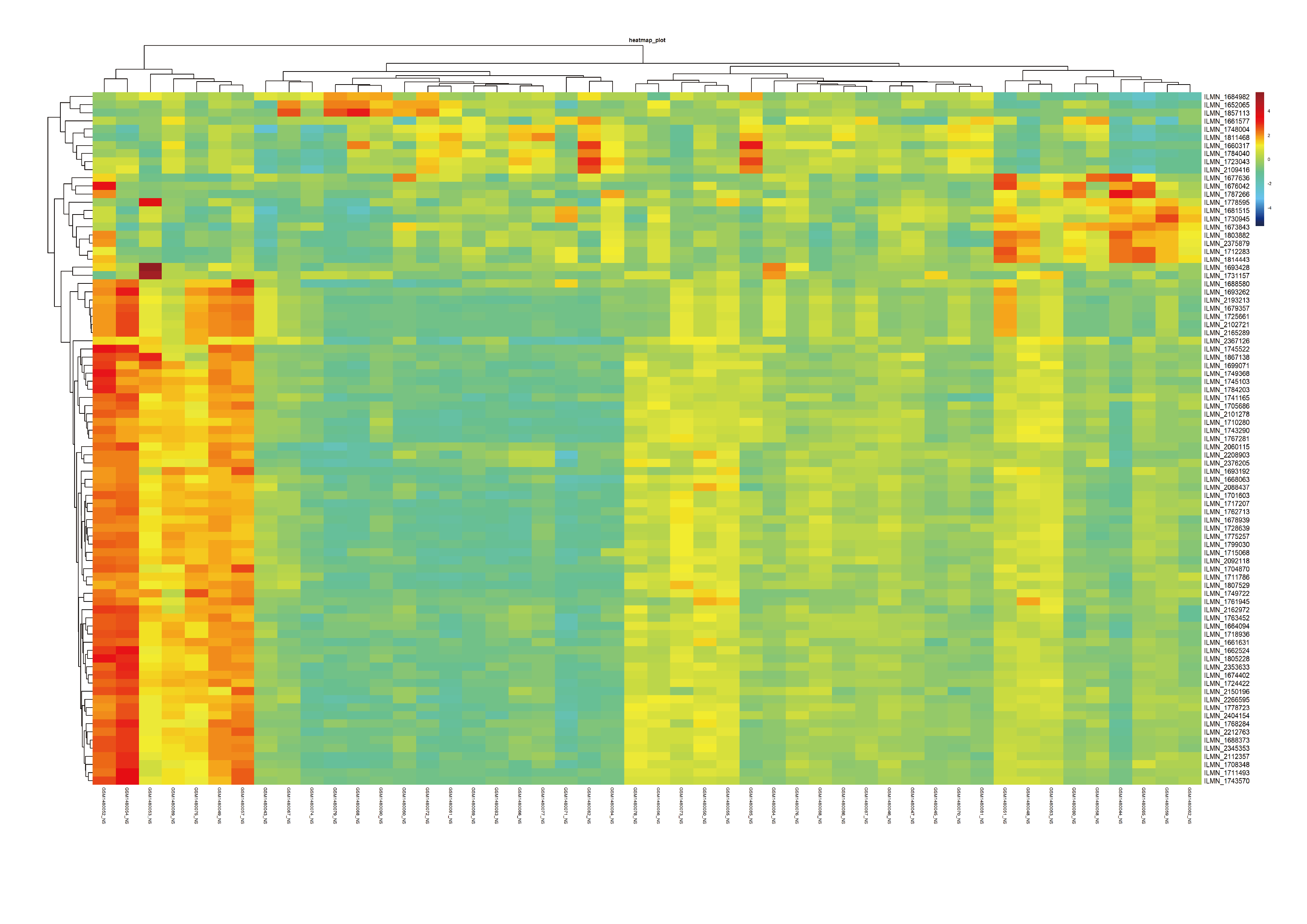

Supplement: Supplementary file 1 — Additional file 1: Fig. S1. The heatmap shows the distribution of differentially expressed genes. [file 12859_2022_5086_MOESM1_ESM.tif]
